# Nasal nitric oxide is useful in an Australian Primary Ciliary Dyskinesia clinic

**DOI:** 10.1186/2046-2530-1-S1-P7

**Published:** 2012-11-16

**Authors:** L Morgan, LJ Buddle, PG Rogers, LM Seccombe, KA MacKenney, LJ Hughes

**Affiliations:** 1 Concord Repatriation General Hospital, Sydney, Australia

## Introduction

Nasal nitric oxide (nNO) has a well-established role as an indirect discriminative marker between patients with and without PCD. All referral services expect to confirm this diagnosis in a minority whereas all referred patients face the inconvenience of travel to an expert centre. a triage test (such as nNO) could reduce this inconvenience and increase the efficiency of the service.

## Aim

To evaluate the utility of nNO for risk stratification within an Australian University Hospital ciliary function service.

## Methods

96 patients with unexplained recurrent respiratory tract infections referred May 2009 and July 2011, underwent nasal brush biopsy with examination of ciliary beating by light microscopy, ciliary beat frequency (CBF) measurement using photometry and EM examination of ciliary ultrastructure. Those patients > 5yrs underwent nNO assessment using a single breath exhalation technique prior to nasal brushing. Healthy volunteers were recruited for nNO measurements.

## Results

96 had ciliary brushing for CBF and EM. 52 were > 5yrs and also had nNO measurements. 13 confirmed PCD patients 21(8) years [mean(SD)] and 39 non-PCD patients 25(18) years plus 57 healthy subjects 27(17) years were included in the analysis. nNO for the PCD group was 51(48) ppb compared to non-PCD 732(318) ppb (p<0.001) and normals group 833(273) ppb (p<0.001). A cutoff of nNO < 300 ppb has a PPV 93%, NPV 100%, sensitivity 100%, specificity 97% for PCD.

## Conclusions

Compared with Normals and non-PCD, patients with PCD have very low levels of nNO. Australians without PCD have nNO levels similar to other reported series. nNO measurements > 300ppb have excellent negative predictive value and could be used to stratify expensive and distant referral for comprehensive ciliary structure and function analysis.

